# Robust Framework for PET Image Reconstruction Incorporating System and Measurement Uncertainties

**DOI:** 10.1371/journal.pone.0032224

**Published:** 2012-03-12

**Authors:** Huafeng Liu, Song Wang, Fei Gao, Yi Tian, Wufan Chen, Zhenghui Hu, Pengcheng Shi

**Affiliations:** 1 State Key Laboratory of Modern Optical Instrumentation, Department of Optical Engineering, Zhejiang University, Hangzhou, China; 2 B. Thomas Golisano College of Computing and Information Sciences, Rochester Institute of Technology, Rochester, United States of America; 3 School of Biomedical Engineering, Southern Medical University, Guangzhou, China; Banner Alzheimer's Institute, United States of America

## Abstract

In Positron Emission Tomography (PET), an *optimal* estimate of the radioactivity concentration is obtained from the measured emission data under certain criteria. So far, all the well-known statistical reconstruction algorithms require *exactly* known system probability matrix *a priori*, and the quality of such system model largely determines the quality of the reconstructed images. In this paper, we propose an algorithm for PET image reconstruction for the real world case where the PET system model is subject to uncertainties. The method counts PET reconstruction as a regularization problem and the image estimation is achieved by means of an uncertainty weighted least squares framework. The performance of our work is evaluated with the Shepp-Logan simulated and real phantom data, which demonstrates significant improvements in image quality over the least squares reconstruction efforts.

## Introduction

Positron Emission Tomography (PET) is one of the most important medical imaging modality which provides *in vivo* functional information of biological organs. It utilizes the idea of injecting chemical compounds tagged with positron emitting isotopes into a body to acquire complete coincidence data, which records the concentration information of the isotope distributions at specific locations within the body. The radioactivity images are then reconstructed based on these photon counting measurements.

The reconstruction of isotope concentration distribution is an ill-posed inverse problem. Most of current approaches to tackle this problem can be classified into two general categories, namely the analytical methods, which rely on the inversion of Radon transform, and the iterative approaches, which are based on a statistical description of the physical problem [1]–[6]. Because of the random nature of the radioactive disintegration, the tomographic data are noisy, and therefore it is straight-forward to regard PET reconstruction as a statistical estimation problem. Such approaches, when reconstructing PET images, need to introduce modeling of the data statistics and to make use of some prior information about the PET imaging system (often referred to as system probability matrix, which represents the probability that an emission event will be detected). For instance, Poisson/Gaussian assumptions on photon counting measurement data may be employed to deal with measurement uncertainties, thus constraining the solution space of reconstruction problem in maximum likelihood/least squares based frameworks [7]–[11].

However, so far, the common feature of all statistical-based methods for PET image reconstruction is that the system response model is assumed to be exactly known *a priori*. In real situations, however, it is almost impossible to have the *exact* system model information because real imaging systems are subject to a number of complicated physical effects (such as positron range, photon emission angle, detector sensitivity normalization factors, intercrystal scatter et al.) [12]–[14]. On the other hand, it has been acknowledged that the quality of the system model largely determines the quality of the final reconstructed images [15]–[17], and the importance of incorporating PET system uncertainties into the reconstruction framework is well recognized yet seldom addressed [14], [14], [18]–[21].

In this paper, we investigate the application of the uncertainty weighted least squares principle to PET image reconstruction. Our algorithm, which is based on a min-max formulation, allows the simultaneous incorporation of system model and measurement statistical uncertainties, thus providing a more robust and accurate solution.

## Methods

### PET System and Measurement Modeling

In the PET measurement, initially, when a positron-emitting nuclide decays in the body, the nucleus rids of itself of excess positive charge by emitting a positron, which almost immediately loses its energy by collisions in the surrounding tissues and then combines with an electron and annihilates. Two back-to-back gamma rays of equal energy are then generated. These photon pairs can be detected externally by two opposite detectors using a coincidence technique, forming a coincidence event. These acquired coincidence data record the concentration information of the isotope distributions at specific locations within the body. However, in reality, the coincidence events may also include those that the two gamma rays originating from two unrelated position annihilations are detected within the coincidence resolving time and those that the annihilation photons have an interaction of Compton scattering and lose their directional and energy information before they arrive to the detector system. The former are referred as random coincidences and the latter are called scattered coincidences. To recover a reasonable image, random and scattered photon pairs should be subtracted from coincidence events.

In practice, the emission sinogram data 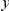, collected in 2-D mode, is a 2-D projection matrix by scanning all detector bins at each angle. The 2-D projection matrix can be transformed into a 1-D vector in the lexicographic ordering. So 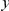 is a 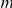 by 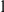 vector with 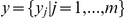 and 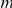 is the total number of bins of the system. 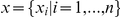 is a 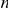 by 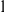 vector with 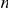 the total number of image voxels, which represents the unknown radioactivity of emission object in voxel 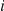. The relationship between the projection data and emission object is given through an affine transform:
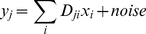
(1)or in matrix form:
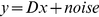
(2)where 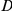 is the 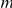 by 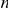 system matrix that gives the probability of a photon emitted from voxel 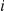 being detected in projection bin 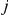. The value of detection probability matrix 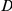 depends on various factors: such as the geometry of the detection system, detector efficiency, attenuation effects, dead time correction factors, and the scattering of photons from one crystal to another.

It is typically difficult or even impossible to obtain an *ideal* known 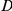 matrix for any real world situation. The errors of system model are broadly classified into two groups - deterministic and statistical [12]. The deterministic errors arise because of the well-known ambiguities (e.g. the geometric ambiguity, attenuation, positron range) presented in the formulation of the model. The statistical errors are results of the random nature of photon detecting, i.e. statistical variations in detector-pair sensitivity, temporo-spatially variant response of the detector caused by the combined effects of intercrystal penetration, cross-talk and the statistical fluctuations in the photomulitplier tube [14], [15], [21]. Here, the goal of our work is to recover the unknown activity distribution 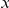 based on the *noisy measurements* 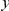 and the *system model* 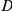 with *random errors*.

### Objective Function Formulation

A way to solve the reconstruction problem is to use the statistical principles, where the objective function is:
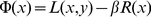
(3)with 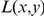 being the log-likelihood, 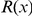 denoting a regularizing penalty term and 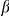 is a hyperparameter that controls the resolution of the reconstructed image. Then the focus of PET imaging becomes to estimate the isotope concentration 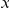 from the noisy measurement data 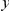 such that 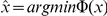. Please note here, in the statistical image reconstruction algorithms, the system matrix 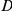 links a tomographic image with the measurements. While the ideal 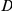 is almost impossible to obtain, the performance of estimators designed without considering these uncertainties can be severely degraded and sometimes even unacceptable(such as for small animal PET imaging). For example, if the actual system matrices were 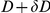, until now, all aforementioned works are based on 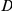 alone, without taking the existence of 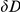 into account. And this inexactness may seriously affect the accuracy and reliability of the estimation results. Here, we introduce an uncertainty weighted least square framework which considers the statistical variations of the system model 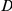 and the measurement data 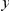.

In order to handle the uncertainty issues of the system and the measurements, a min-max cost function formulation can be adopted to achieve robust solution [22] for (2):
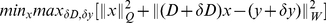
(4)where the notation 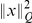 is defined as the square of the weighted 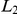 norm of 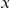 (by constraints 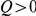), i.e. 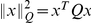. 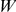 is a weighting matrix.

Directly solving (4) will take too much storage space and computational time. Similar to the penalized weighted method proposed by Fessler [9], we have adopted an iterative algorithm to get the convergent solution of the uncertainty penalized weighted least squares (UPWLS, or called robust least squares, RLS) framework based on state space description of PET imaging. In the following section, we will derive the UPWLS (RLS) formulation with Table 1 giving the notation of related symbols and abbreviations.

**Table 1:** Definitions of symbols and abbreviations.

*Symbols*	
x	radioactivity distribution
y	emission sinogram data
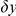	perturbation of sinogram data
m	total number of bins in the system
n	the total number of image voxels
D	system matrix
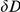	perturbation of system matrix
Q	penalty matrix
W	weighting matrix
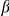	smoothing parameter
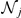	neighborhood of the 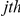 pixel
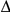	norm-bounded uncertainties contraction
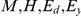	constant constraint for norm-bounded uncertainties
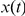	state variable in state space model
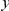	measurement variable in state space model
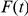	state transition matrix
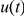	model uncertainties in state space description
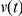	measurement noise in state space description
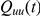	covariance of model uncertainty 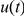
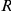	covariance of measurement noise 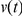
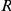	covariance of time-independent measurement noise 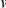

*Abbreviations*	
PET	Positron Emission Tomography
PWLS	Penalized Weighted Least Squares
UPWLS	Uncertainty Penalized Weighted Least Squares
SOR	Successive Over-Relaxation
CG	Conjugate Gradient
FBP	Filtered Backprojection
EM	Expectation Maximization

### UPWLS Framework for PET Imaging

#### Deterministic Interpretation of PET Imaging

Here, the stationary PET inverse problem is considered. In static imaging case the concentration 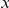 is assumed to be nonvarying, which means
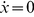
(5)Discretizing it, we have
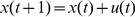
(6)Please note here, 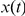 means activity distribution after 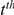 time (updated) step and 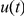 represents the uncertainties of the state updating process. Together with PET observations, the PET imaging can thus be interpreted in state space description as:
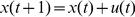
(7)

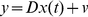
(8)where 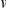 models the *measurement noise*. Here we treat 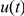 and 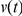 as random variables with mean and covariance matrix as
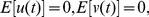
(9)

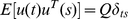
(10)

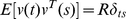
(11)


#### Uncertain State Space Model for PET imaging

Now, let us consider the case of uncertainty in matrix 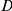, the state space equations in the subsection above could be rewritten with the uncertainty item 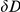 as:
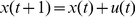
(12)

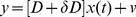
(13)Here we introduce the norm-bounded uncertainty model [23] for the system uncertainties as
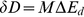
(14)where 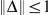 is an arbitrary contraction, 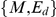 are known real constant matrices of proper dimensions that specify how the uncertain parameters in 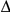 enter the matrix 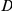. The case of accurate models could be obtained by setting 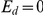.

For above uncertain state space description, once having a *priori* estimate 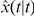 together with variance 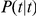 for the state 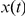 (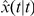 is the estimation of 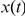 at step 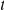, with corresponding variances 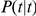.), and giving measurements 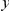, updating the estimate of state variable from 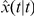 to 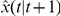 could be realized by solving
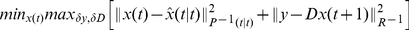
(15)The estimation criterion is to minimize the worst possible effects of the disturbances on the signal estimation errors, which ensures that if the disturbances are small, the estimation errors will be as small as possible. This characteristic makes the method to be appropriate for some practical problems that disturbanfces exist in both system and measurements.

### UPWLS Solution for PET Image Reconstruction

To solve the objective function (15), we model uncertainties in system and measurement with a norm-bounded structure [23] as
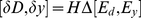
(16)where 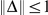 is an arbitrary contraction, 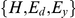 are known real constant matrices of proper dimensions that specify how the uncertain parameters in 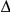 enter the matrices 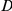 and 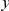.

Consider about the min-max cost function (15), by setting a corresponding relationship as
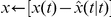


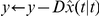


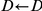


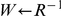


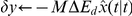


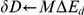


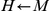


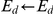


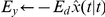


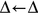
(17)it is easy to get the objective function:
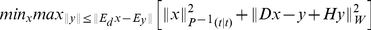
(18)Which is just the equivalent form of (4) under the uncertainty model (16). Here 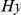 is defined as
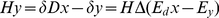
(19)According to the result published in 2001 [22], we can obtain the following unique solution for (18) by solving:
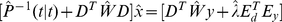
(20)

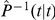
 and 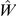 could be defined as
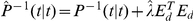


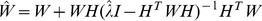
(21)where parameter 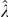 could be calculated by minimizing 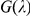 defined in Appendix S1 over the interval 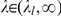 (further detail about how to define 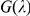 and how to decide 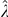 will be given in Appendix S1) with
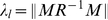
(22)


In practice, we can choose a reasonable approximation for 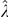, which is to set it equal to a multiple of its low bound 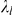 as [22]

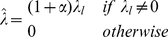
(23)where 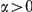 is designed by the user, that could be chosen to be time variant as well.

For any determined 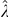 with the corresponding relationship in equation (17), if let 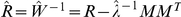, equation (20) could be written as
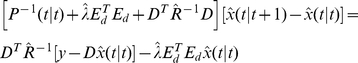
(24)By setting
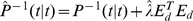
(25)

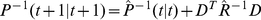
(26)

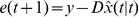
(27)

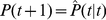
(28)and let
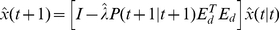
(29)we can get
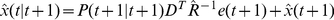
(30)Further more, we can obtain a form for iteration of 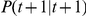 if let
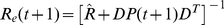
(31)that is
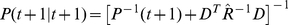
(32)

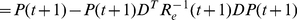
(33)According to the conclusions above, an iterative process of UPWLS estimation for the state space based PET reconstruction would be summarized as(Please see Appendix S2 for a brief derivation):

**Given the uncertain model:**

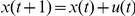


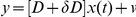


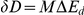
with known 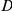, 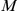, 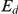 and covariance matrix 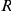, as defined in (12)–(14).

**Initialization:** with given initial state estimation 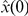 ( the initial activity distributions are zero or set to the results from filtered back projection method for faster convergence), covariance matrix 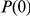 and measurement 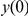, here, P(0) is initialized with the inversion of penalty term Q, the initial penalty Q we used is a simple quadratic smoothness penalty. Set 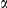 to get 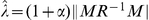 and calculate 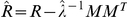, then make initialization according to (32)
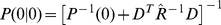
(34)

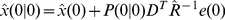
(35)

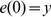
(36)


**Update step from** *a priori* **estimation** 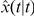 **and** 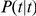 **:**

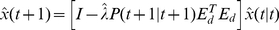
(37)

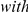


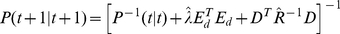
(38)

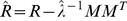
(39)


**Correction step with given measurement** 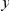 **:**

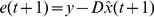
(40)

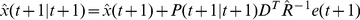
(41)


## Results and Discussion

### Digital Phantom Data

**Figure 1:**
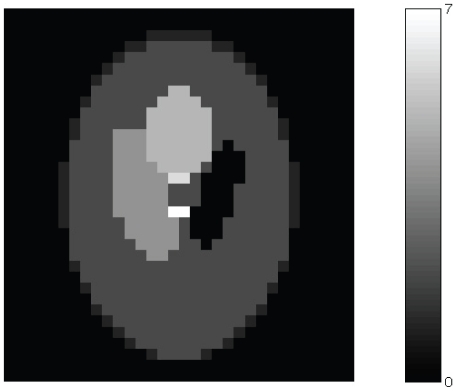
Digital Shepp-Logan phantom used in the experiments.

The well-known and widely used Shepp-Logan synthetic emission phantom (Fig. 1) with known radioactivity concentrations has been used to evaluate our algorithm. To generate realistic data, we simulate the emission coincidence events during prompt windows and delayed windows respectively. The prompt data has to be modified to subtract the effects of the random events. Taking these effects into account, the measured sinogram 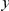 is created based on the equation:
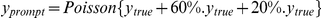


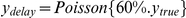


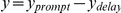
Here, we model the random and scatter events to be uniform field of 60 percents and 20 percents respectively. 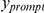 is the number of coincident photon pairs collected in the prompt windows, while 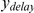 is the number of coincident photon pairs collected in the delay windows. The total photon counts are set to be 100K. Then we generate 50 realizations of pseudorandom emission measurements independently. In order to investigate how the noise in the system matrix affects the reconstructed image, we generate a noisy system matrix 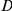 through changing the factors related to attenuation effects with the mean relative error in the range of 0%–15%, where the error is defined by
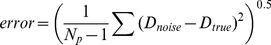
(42)with 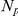 is the total number of pixels. Finally, each noisy sinogram is reconstructed with three different algorithms: the popular EM and PWLS+CG (where the penalty term is a commonly used standard quadratic smoothing penalty) [9], and the proposed UPWLS method. These processes are executed iteratively until it meets the convergence criterion, which is defined by two consecutive normalized errors 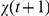 and 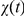 through 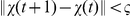 with 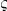 being a small constant, and 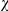 defines the normalized error between the estimated and the exact value with
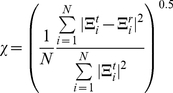
(43)where 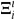 is the estimated value, 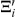 is the corresponding true value, and 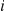 indicates the pixel.

**Figure 2:**
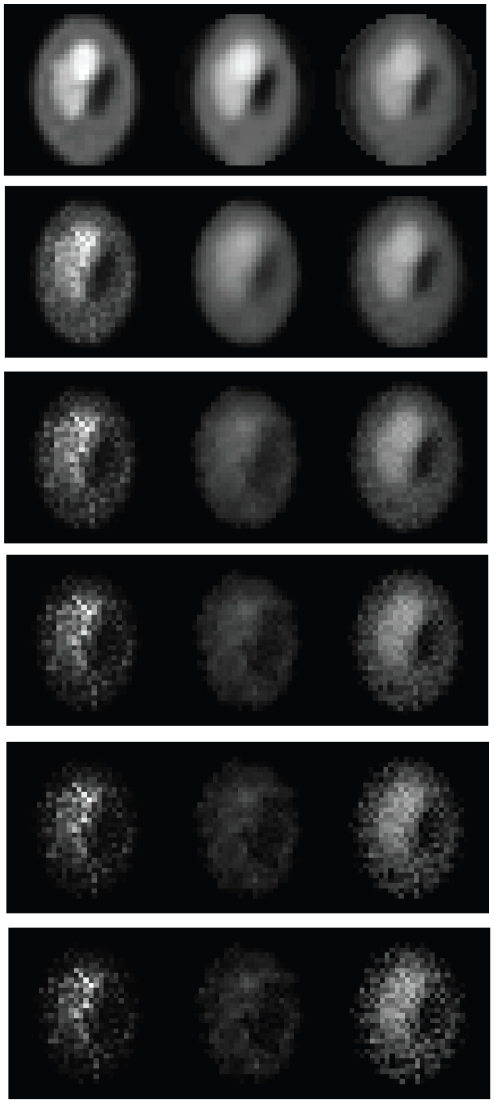
From top to bottom: The mean of Shepp-Logan phantom images reconstructed based on noisy system matrix with relative error 0%, 3%, 6%, 9%, 12%, and 15%. (From left to right: EM results, PWLS results, UPWLS results.).

**Figure 3:**
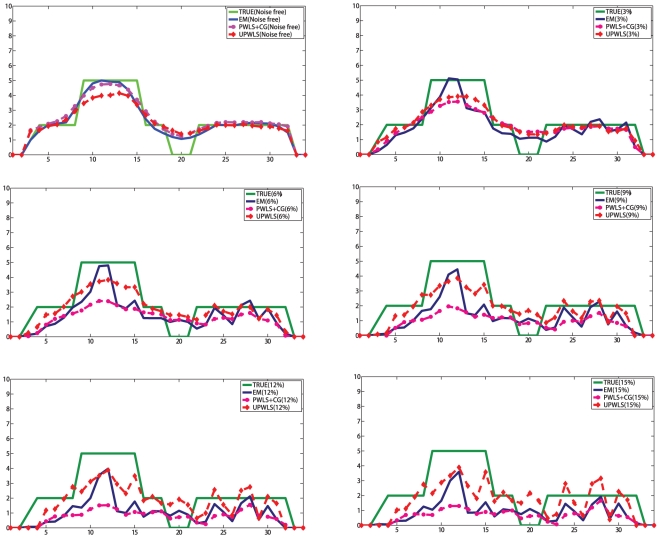
Horizontal profiles through sample mean of estimators based on noisy system matrix with relative error 0%, 3%, 6%, 9%, 12% and 15%.

The images of the mean pixel values obtained by the three algorithms (EM [7], PWLS+CG [9], [10], UPWLS) based on noisy system matrix are shown in Fig. 2, while the horizontal profiles of the 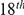 row through the sample mean are plotted in Fig. 3. The figures show obviously that the PWLS+CG results have large bias, while the UPWLS framework seems free of such a bias.

A detailed statistical analysis on the estimation results against the ground truth phantom map is performed. Let 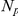 be the total number of pixels and 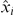 be the final reconstruction result of pixel 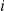 respectively, and 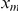 be the mean value of the ground truth through all the pixels, then we have the following error definitions:
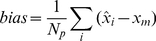
(44)

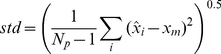
(45)


The bias and variance of errors are averaged over the 50 reconstructions to give the estimates bias and variance at different noise levels which are summarized in Table 2. EM and PWLS perform well in noise-free case, but degradation of the image quality is observed in the noisy system model. The UPWLS framework results demonstrate the bias and standard variation remain more stable over the changing system matrix, which can be observed more clearly by percentage. For example, the bias is improved by 25.21% and 35.48% in average over EM and PWLS algorithms, and the standard deviation is improved by 26.63% and 23.56% in average over EM and PWLS algorithms, respectively, for the case with model error of 6%. Overall, these figures and results illustrate that it is possible that small noise errors lead to large estimation errors for traditional methods. On the other hand, very stable results are obtained with UPWLS framework, showing its desired robustness for real-world problems.

**Table 2:** Comparative studies of estimated activity distribution on synthetic data.

Noise Level	EM	PWLS+CG	UPWLS
0%	0.18961 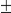 0.38458	0.22543 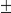 0.40019	0.31124 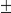 0.53637
3%	0.35910 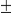 0.61881	0.34407 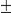 0.58749	0.32570 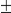 0.54911
6%	0.51466 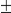 0.85399	0.59663 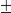 0.94242	0.38493 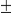 0.62611
9%	0.61897 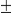 1.01270	0.74372 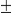 1.15510	0.48881 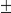 0.75809
12%	0.69197 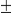 1.12480	0.82508 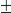 1.28050	0.58294 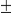 0.89329
15%	0.74476 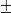 1.20850	0.87524 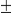 1.35990	0.66465 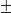 1.01970

Each data cell represents reconstruction error: bias 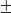 std.

### Real Phantom Data

**Figure 4:**
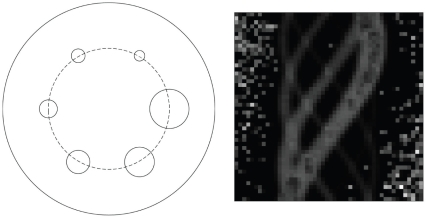
The geometry of real phantom, emission sinogram obtained by the SHR-22000.

**Figure 5:**
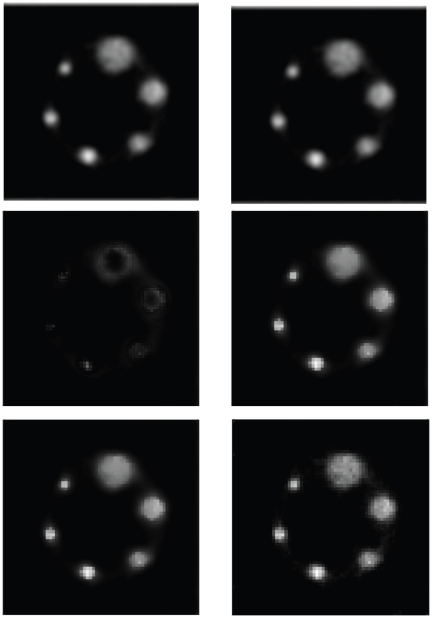
The reconstructed radioactivity map using the EM(top), PWLS(middle), and UPWLS(bottom) method in 4 iterations (left) and 12 iterations(right).

**Figure 6:**
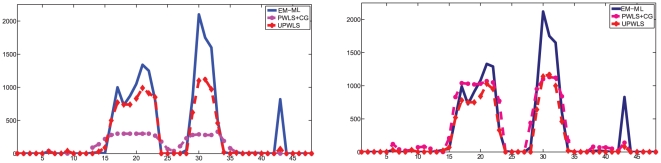
Vertical profiles through reconstructed images: 4 iterations (left) and 12 iterations(right).

The real data set used in this study was acquired on Hamamatsu SHR-22000 scanner [24] using a 6-spheres phantom, which is usually employed to measure the recovery coefficients. The SHR-22000 scanner is designed as a whole body imaging system. It has a 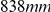 detector ring diameter with a patient aperture of 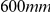, an axial field of view (FOV) of 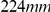, operates in 2D or 3D mode. For the phantom, there are six circular regions of different diameters. These sphere objects have diameters of 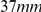, 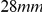, 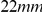, 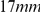, 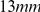, 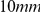 and are inserted in a circular cylinder with diameter of 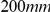 corresponding to a volume of 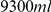, as shown in Fig. 4. The phantom filled with pure water was located at the center of both transaxial and axial FOV in the scanner using the patient bed. Transmission data were acquired in 2-D and prompt-delayed coincidence model using rotating 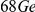 rod sources with total activity around 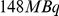. A long blank scan was first acquired for 60 minutes. We injected F-18 concentration with initial activity of 107.92 kBq/ml into the six spheres. A 120-minutes scan was then performed. Fig. 4 shows the sinograms obtained from the emission scan. Here, system matrix 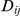 is computed by with a single ray approximation model, which can be approximately viewed as the length of intersection between the 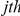 pixel and the 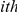 projection ray, i.e. 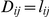. Longer 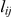 indicates larger detection sensitivity.The random events have been removed by utilizing delayed window coincidence technique. Conventional EM methods, PWLS and our algorithm as described in the previous section have been applied to recover images from the noisy data as shown in Fig. 5. Along with the vertical profiles (34# col), as shown in Fig. 6, it is evident that UPWLS results are faster, more stable, and can achieve more robust convergence of the estimates. Since PWLS converges more slowly, a longer computation time is thus expected. In a further quantitative analysis of the algorithms, the mean concentration values with standard derivation of the estimates are summarized in Table 3. It can seen that the variance performance for the UPWLS is better than EM, while the mean performance is slightly worse. Two reasons lead to such result: (1) The parameters used in UPWLS are not yet optimal; (2) The UPWLS does not incorporate the Poisson model of the measurement data while EM does.

**Table 3:** Quantitative values measured from the six-sphere phantom.

True value(kBq/ml)	EM (kBq/ml)	PWLS+CG (kBq/ml)	UPWLS (kBq/ml)
107.92	114.18 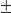 12.42	120.04 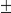 14.74	116.91 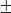 9.31

### Discussion

There are many different approaches for PET image reconstruction from projections, most of which are based on exactly known system model or system matrix. Several attempts were made to tackle this problem based on a specific type of modelling error, such as positron range, non-colinearity of the photon pair, and depth of interaction effect et al. Unlike these existing efforts that are limited to single type of modelling error, we propose an UPWLS method to handle statistical uncertainties of the system.

The P (0), 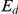, and R matrices that are used in the UPWLS framework are chosen according to the confidence measurements on state 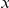, system model and measurement noise respectively. For example, if we know that the noise in the system model is smaller than the measurement noise, we should make the 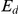 matrix smaller than the 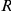 matrix, which de-emphasizes the importance of the uncertainties of system model relative to the measurement noise. Further, we believe that any prior knowledge of the system model and measurement data should enable us to achieve higher estimation efficiency and more robust results.

In our current implementation, the parameters are set to some empirically fixed values in all experiments: 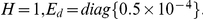 Ideally, these parameters could be adaptively updated during the estimation process. The simulation experiments are designed to show the robustness and accuracy of the proposed method, and the physical phantom experiment is used to show its efficiency and accuracy for the real world problem. Overall, our experiment results reveal that it is possible that small noise errors in system matrix may lead to large estimation errors for exactly known model-based schemes, while the UPWLS framework produces consistent results even with highly noisy system matrix, which promises robustness for real-world problems.

On the other hand, current methods requires huge storage and expensive computation as today's PET scanners have a large set of detector pairs and the inversion of the system matrix. With the method mentioned in the Appendix S3, we are able to get the inversions faster and more accurate. Furthermore, another fast algorithm, called fast state space filter has been developed. The most interesting thing is that our framework naturally allows the combination of the reconstruction process with the data acquisition task.

As a continuation of this work, based on the tracer kinematics equation coming from compartment model, we can recover dynamic changes of tracer density in a continuous time domain for dynamic PET with an uncertain system model.

Detailed investigations on these issues are underway.

### Conclusion

In this paper, we have developed an uncertainty weighted least squares framework for the estimation of activity distribution from PET measured data. The approach enables us to incorporate the system uncertainties into PET image reconstruction, thus providing more robust and stable results. Analytical and experimental results with Shepp-Logan simulation phantom data and real PET measurements demonstrate the power of the proposed method.

## Supporting Information

Appendix S1 **Derivations for UPWLS solution.**
(PDF)

Appendix S2 **The iterative expression for state variable and variance.**
(PDF)

Appendix S3 **Inversion of a large sparse matrix - GMRES(Generalized Minimal Residual) method.**
(PDF)
