# Supplementary material for: Robust Framework for PET Image Reconstruction Incorporating System and Measurement Uncertainties
Source: PLoS One. 2012 Mar 12;7(3):e32224. doi: 10.1371/journal.pone.0032224 (PMC3299650; doi:10.1371/journal.pone.0032224)
Supplement: Appendix S1 — Derivations for UPWLS solution. (PDF) [file pone.0032224.s001.pdf]

# Appendix S1: Derivations for UPWLS solution.

In section of methods, we finally derived the iterative updating or state variable and its variance by using a parameter $\lambda$ as determined number $\hat{\lambda}$. Here we will show more details about the definition of $\lambda$ and related calculation.

For the min-max cost function (18) we used for solving UPWLS, we can obtain the unique solution[22] as

$$\hat{x} = [\hat{Q} + D^T \hat{W} D]^{-1} [D^T \hat{W} y + \hat{\lambda} E_d^T E_y] \tag{1}$$

with

$$\begin{aligned} \hat{Q} &= Q + \hat{\lambda} E_d^T E_d & (2) \\ \hat{W} &= W + WH(\hat{\lambda} I - H^T W H)^{-1} H^T W & (3) \end{aligned}$$

By applying the corresponding relationship in (17), we could have

$$\begin{aligned} \hat{Q} &= P^{-1}(t|t) + \hat{\lambda} E_d^T E_d & (4) \\ \hat{W} &= (W^{-1} - \hat{\lambda}^{-1} H H^T)^{-1} & \\ &= (R - \hat{\lambda}^{-1} M M^T)^{-1} & (5) \end{aligned}$$

just the result in (21).

Here we defined $\hat{\lambda}$ as a nonnegative scalar parameter, which is determined from:

$$\begin{aligned} \hat{\lambda} &= argmin G(\lambda) & (6) \\ \lambda &\geq \| H^T W H \| & (7) \end{aligned}$$

The function $G(\lambda)$ is given by:

$$G(\lambda) = \|x(\lambda)\|_Q^2 + \lambda \|E_d x(\lambda) - E_y\|^2 + \|Dx(\lambda) - y\|_{W(\lambda)}^2 \tag{8}$$

with

$$\begin{aligned} W(\lambda) &= W + WH(\lambda I - H^T W H)^{-1} H^T W & (9) \\ Q(\lambda) &= Q + \lambda E_d^T E_d & (10) \\ x(\lambda) &= [Q(\lambda) + D^T W(\lambda) D]^{-1} [D^T W(\lambda) y + \lambda E_d^T E_y] & (11) \end{aligned}$$

Related discussion on the solution to the uncertain system and the detail about selection of parameter $\lambda$ can be found in [22].
