# Supplementary material for: Robust Framework for PET Image Reconstruction Incorporating System and Measurement Uncertainties
Source: PLoS One. 2012 Mar 12;7(3):e32224. doi: 10.1371/journal.pone.0032224 (PMC3299650; doi:10.1371/journal.pone.0032224)
Supplement: Appendix S2 — The iterative expression for state variable and variance. (PDF) [file pone.0032224.s002.pdf]

# Appendix S2: The iterative expression for state variable and variance.

Here we will give a brief deviation about the iterative process.

After given the a *priori* estimation $\hat{x}(t|t)$ for $x(t)$, and $P(t|t)$, a measurement update $\hat{x}(t|t+1)$ by giving $y(t+1)$ could be calculated if applying the corresponding relations (17) on (20) with the definition in (21) and determined $\hat{\lambda}$. Then the left side of equation (20) becomes

$$\left[\hat{Q} + D^T\hat{W}D\right]\hat{x} = \left[P^{-1}(t|t) + \hat{\lambda}E_d^T E_d + D^T\hat{W}D\right][\hat{x}(t|t+1) - \hat{x}(t|t)] \tag{1}$$

and the right side becomes

$$D^T\hat{W}y + \hat{\lambda}E_d^T E_y = D^T\hat{W}[y(t+1) - D\hat{x}(t|t)] - \hat{\lambda}E_d^T E_d\hat{x}(t|t) \tag{2}$$

where

$$\hat{W} = \left(R - \hat{\lambda}^{-1}MM^T\right)^{-1} \tag{3}$$

If let $\hat{R} = \hat{W}^{-1} = R - \hat{\lambda}^{-1}MM^T$, we can get

$$\left[P^{-1}(t|t) + \hat{\lambda}E_d^T E_d + D^T\hat{R}^{-1}D\right][\hat{x}(t|t+1) - \hat{x}(t|t)] = D^T\hat{R}^{-1}[y(t+1) - D\hat{x}(t|t)] - \hat{\lambda}E_d^T E_d\hat{x}(t|t)$$

By setting

$$\begin{aligned}\hat{P}^{-1}(t|t) &= P^{-1}(t|t) + \hat{\lambda}E_d^T E_d \\ P^{-1}(t+1|t+1) &= \hat{P}^{-1}(t|t) + D^T\hat{R}^{-1}D\end{aligned}$$

we will have the time update $\hat{x}(t+1)$ from $\hat{x}(t|t)$ as

$$\hat{x}(t+1) = \left[I - \hat{\lambda}P(t+1|t+1)E_d^T E_d\right]\hat{x}(t|t)$$

and then if we set

$$\begin{aligned}e(t+1) &= y(t+1) - D\hat{x}(t|t) \\ P(t+1) &= \hat{P}(t|t)\end{aligned}$$

the measurement update $\hat{x}(t|t+1)$ could be

$$\hat{x}(t|t+1) = P(t+1|t+1)D^T\hat{R}^{-1}e(t+1) + \hat{x}(t+1)$$

here we can obtain a form for iteration of $P(t+1|t+1)$ if let

$$\begin{aligned}P(t+1) &= \hat{P}(t|t) = \left[P^{-1}(t|t) + \hat{\lambda}E_d^T E_d\right]^{-1} \\ R_e(t+1) &= \left[\hat{R} + DP(t+1)D^T\right]^{-1}\end{aligned}$$

that is

$$\begin{aligned}P(t+1|t+1) &= \left[P^{-1}(t+1) + D^T\hat{R}^{-1}D\right]^{-1} \\ &= P(t+1) - P(t+1)D^T R_e^{-1}(t+1)DP(t+1)\end{aligned}$$

For the final state update, since for static reconstruction problem we have the state equation as (12), we can get

$$\hat{x}(t+1|t+1) = \hat{x}(t|t+1) = P(t+1|t+1)D^T\hat{R}^{-1}e(t+1) + \hat{x}(t+1) \tag{4}$$

then an iterative process from known $\{\hat{x}(t|t), P(t|t)\}$ and $\{y(t+1)\}$ to $\{\hat{x}(t+1|t+1)$ was given as above.
