# Supplementary material for: Robust Framework for PET Image Reconstruction Incorporating System and Measurement Uncertainties
Source: PLoS One. 2012 Mar 12;7(3):e32224. doi: 10.1371/journal.pone.0032224 (PMC3299650; doi:10.1371/journal.pone.0032224)
Supplement: Appendix S3 — Inversion of a large sparse matrix - GMRES(Generalized Minimal Residual) method. (PDF) [file pone.0032224.s003.pdf]

Appendix S3: Inversion of a large sparse matrix - GMRES(Generalized Minimal Residual) method.

For the inversion of a large sparse matrix, we use the procedure of incomplete LU factorization as precondition and GMRES(Generalized Minimal Residual) method[25].

The LU factorization expresses a matrix as the product of two essentially triangular matrices, one of them a permutation of a lower triangular matrix and the other an upper triangular matrix, sometimes is called the LR factorization. The GMRES (Generalized Minimal Residual) method is used successfully to solve large nonsymmetric linear systems of equations. Here, LU factorization is used to speed up the calculation, GMRES method can be applied to solve very large sparse matrix.

The inversion of the matrix A can be expressed as

$$AX = I \tag{1}$$

with $A, X, I \in \mathbb{R}^{n\times n}$, n is the dimension of matrix A and I is an identity matrix, thus X is equal to the inversion of matrix A. With the incomplete LU factorization of matrix A, equation (1) can be rewritten as

$$LUX = I \tag{2}$$

with $A = LU$, and I can be treated as a 1 by n vector. So equation (2) could be written as

$$LUX = [e_1, e_2, \cdots, e_n] \tag{3}$$

equation (3) can be treated as a group of linear equations, they are calculated using GMRES method first to get the matrix UX, and then with the matrix UX, GMRES method is applied again to get the matrix X, that is the inversion of matrix A.
